# Stomach-specific c-Myc overexpression drives gastric adenoma in mice through AKT/mammalian target of rapamycin signaling

**DOI:** 10.17305/bjbms.2020.4978

**Published:** 2021-08

**Authors:** Jing Liu, Wenxin Feng, Min Liu, Hanyu Rao, Xiaoxue Li, Yan Teng, Xiao Yang, Jin Xu, Wei-Qiang Gao, Li Li

**Affiliations:** 1 State Key Laboratory of Oncogenes and Related Genes, Renji-Med X Clinical Stem Cell Research Center, Ren Ji Hospital, School of Medicine and School of Biomedical Engineering, Shanghai Jiao Tong University, Shanghai, China; 2 School of Biomedical Engineering and Med-X Research Institute, Shanghai Jiao Tong University, Shanghai, China; 3 State Key Laboratory of Proteomics, Beijing Proteome Research Center, National Center for Protein Sciences, Beijing Institute of Lifeomics, Beijing, China

**Keywords:** c-Myc, gastric adenoma, transgenic, AKT/mammalian target of rapamycin

## Abstract

Gastric cancer (GC) is one of the most common malignant cancers in the world. *c-Myc*, a well-known oncogene, is commonly amplified in many cancers, including GC. However, it is still not completely understood how *c-Myc* functions in GC. Here, we generated a stomach-specific *c-Myc* transgenic mouse model to investigate its role in GC. We found that overexpression of *c-Myc* in *Atp4b^+^* gastric parietal cells could induce gastric adenoma in mice. Mechanistically, *c-Myc* promoted tumorigenesis through the AKT/ mammalian target of rapamycin (mTOR) pathway. Furthermore, AKT inhibitor (MK-2206) or mTOR inhibitor (rapamycin) inhibited the proliferation of *c-Myc* overexpressing GC cell lines and the initiation of gastric tumorigenesis in *c-Myc* transgenic mice. Thus, our findings highlight that gastric tumorigenesis can be induced by *c-Myc* overexpression through activation of the AKT/mTOR pathway.

## INTRODUCTION

Gastric cancer (GC) is one of the most common malignant cancers in the world. It was reported that in 2018, the incidence of GC ranked the fifth in the world, while the mortality rate ranked the third [1]. East Asian countries, including China, have a high incidence of GC, partly due to the high-salt diet. Although the diagnosis means have improved in recent years, the diagnosis of early GC is still poor because of the lack of apparent symptoms. Besides, patients receiving a conventional treatment have a recurrence rate of 50% and a 5-year survival rate of 20% [2]. According to the Lauren classification [3], GC can be classified into two types: Intestinal type and diffuse type, of which the intestinal-type gastric cancer accounts for 60%-75% [4]. Gastric carcinogenesis follows a series of precancerous phases, which is called Correa’s cascade [5], including chronic gastritis, atrophic gastritis, intestinal metaplasia (IM), dysplasia, and eventually GC. As the precancerous lesions of GC can last for a long period, it is important to identify the causal drivers for the development of early GC.

Mouse model is commonly used to investigate the pathogenesis of various cancers since the protein-coding genes of mice and human share high similarity [6]. Establishment of mouse models of GC has progressed from chemically induced random mutagenesis, to bacterial-induced dysplasia and to genetically engineered mouse models (GEMMs) [7]. These models have revolutionized our understanding of the effects of diet, bacteria, and genes on gastric carcinogenesis. Of these, GEMMs are proven to be the most useful tool for dissecting the roles that individual genes and signaling pathways play in GC. These models include the introduction of mutations in oncogenes and tumor suppressor gene loci, as well as abnormal expression of signaling factors.

The *c-Myc* is a well-known oncogene involved in various cancers, including GC. Amplification of *c-Myc* in GC has been reported in several studies [8-11]. Gain of *c-Myc* copies (≥3) is linked with late on-set, intestinal-type, advanced tumor stage, and distant metastasis, while *c-Myc* hypomethylation is associated with diffuse-type GC [12]. It is reported that *c-Myc* overexpression is more frequently observed in GC than gene amplification [13,14]. *c-Myc* overexpression was described in over 40% of GC [15]. De Souza et al. observed that 77% of the gastric tumors present significantly increased *c-Myc* mRNA expression, which was associated with deeper tumor extension and metastasis [12,16]. Notably, overexpression of *c-Myc* is more frequently observed in intestinal-type GC than diffuse-type GC [12,17,18] and is associated with malignant progress and poor survival in GC patients [18-20]. MYC protein expression increased progressively from chronic gastritis, IM, dysplasia, early GC to progressive GC [17,18,21,22]. However, whether overexpression of *c-Myc* is sufficient to cause GC remains unclear.

The *c-Myc* plays a crucial role in several cellular functions, such as cell proliferation, differentiation, and cell cycle progression [23]. It was reported that c-*Myc* transcriptionally regulates the expression of TRAP1, which controls primary and metastatic tumor growth [24]. In renal cell carcinoma (RCC), c-*Myc* induces RCC in a glutamine-addicted way [25]. Significant upregulation of *c-MYC* proteins, which is resulted from alterations of the Wnt and Ras pathways, is often seen in 70% colorectal cancer [26-28]. While in GC, it was reported that *BRD4* could promote GC progression through positive regulation of *c-Myc* in transcription and epigenetic levels [29] and knockdown of *c-Myc* could inhibit the growth and proliferation of GC cell lines [30,31]. Liu et al. reported that USP22 promoted GC progression through the activation of *c-Myc/NAMPT/SIRT1*-dependent FOXO1 and YAP signaling pathways [32]. Xu et al. found that KLF5 and MYC could transcriptionally enhance the expression of LINC00346, which was a GC inducer *in vitro* and *in vivo* [33]. Choi et al. reported that YAP/TAZ activation could initiate gastric carcinogenesis through transcriptionally upregulating MYC in the knockout mice for *Lats1* and *Lats2* [34]. There have been several *Myc*-driven mouse models of cancer, including prostate cancer [35] and renal cell carcinoma [25], but not GC, to the best of our knowledge. It was reported that MYC inactivation could induce sustained regression of invasive liver cancer in a MYC transgenic mouse model [36]. Thus, investigating the direct impact of *c-Myc* on GC would be of great interest to uncover new therapies for GC.

In this study, we generated a novel gastric tumor model in which human *c-Myc* is highly expressed in gastric parietal cells to investigate the definite role of *c-Myc* in GC. We present data indicating that these mice developed the phenotypic features of the gastric adenoma, with a step-wise tumorigenic progression from hyperplasia to metaplasia, dysplasia, and finally adenoma in gastric mucosa. Importantly, our findings highlight a mechanism by which gastric adenoma can be induced by stomach-specific *c-Myc* overexpression through activation of the AKT/mammalian target of rapamycin (mTOR) pathway.

## MATERIALS AND METHODS

### Mice

*Atp4b-cre* mice were gifted from Dr. Xiao Yang and described previously [37]. *Myc^fl/fl^* mice were purchased from the Jackson Laboratory and were also described previously [38]. *Atp4b-cre; Myc^fl/+^*, referred as *Atp4b-cre; Myc^OE^* mice were generated by crossing *Atp4b-cre* mice with *Myc^fl/fl^* mice. *Myc^fl/fl^* mice were used as control. Both male and female mice were used for experiments since no difference of sex has been observed. All the mouse strains were generated in a C57BL/6 background and were born and maintained in a specific-pathogen-free (SPF) facility and all experimental procedures were approved by the Animal Ethics Committee of School of Biomedical Engineering and Med-X Research Institute, Shanghai Jiao Tong University. All institutional and national guidelines for the care and use of laboratory animals were followed. Primers for genotyping are shown in Table S1.

**TABLE S1:**
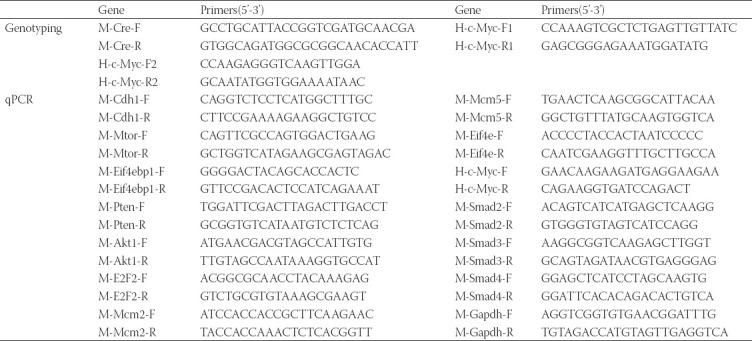
Primers for genotyping and qPCR

### Histology, hematoxylin and eosin (H&E), and immunohistochemistry (IHC) staining

Mice were sacrificed at different ages (12 w, 25 w, and 35 w). Mouse stomachs were then harvested, cut open through the greater curvature, and washed 3 times in phosphate-buffered saline by vigorous shaking. Tissues were fixed in 4% poly-formaldehyde for 24 hours at 4°C, then dehydrated and embedded in paraffin. Sections (5 μm) were cut and stained with H&E. Tumor grades (0-4) were scored according to the previous report [39]. For IHC staining, sections were deparaffinized, rehydrated, subjected to antigen retrieval in citrate buffer, and quenched for endogenous peroxidases with 3% H_2_O_2_. Blocking was performed with 5% BSA for 1 hour at room temperature. The primary antibodies used here were anti-c-MYC (Abcam, ab32072, 1:200), anti-Ki67 (Abcam, ab15580, 1:2000), anti-MUC2 (Santa, sc-515032, 1:200), and anti-MUC5AC (Abcam, ab3649, 1:200). Periodic Acid-Schiff/Alcian Blue (PAS/AB) staining was performed using AB/PAS stain kit (Solarbio, G1285). Staining intensities were calculated using Image J software.

### Western blotting

Tissue and cell lysates were prepared by strong radioimmunoprecipitation assay buffer (Beyotime, P0013B) containing protease inhibitors and supplemented with protein phosphatase inhibitors (mammalian cell entry [MCE]). The proteins were separated by sodium dodecyl sulfate-polyacrylamide gel electrophoresis gels and then transferred to polyvinylidene fluoride membranes (Millipore). The membranes were blocked with 6% skim milk in Tris-Buffered Saline Tween-20 for 1.5 h at room temperature and subsequently incubated with specific primary antibodies overnight at 4°C followed by incubation with secondary antibodies for 1 h. The primary antibodies used in this study were as follows: Anti-flag-tag (cell signaling technology [CST], 14793, 1:1000), anti-c-MYC (Abcam, ab32072, 1:1000), anti-β-Actin (Abmart, P30002, 1:4000), anti-PI3K (CST, 4292, 1:1000), anti-p-PI3K (CST, 4228, 1:1000), anti-AKT (CST, 2920, 1:2000), anti-p-AKT (CST, 4060, 1:2000), anti-mTOR (CST, 2983, 1:1000), anti-p-mTOR (CST, 5536, 1:1000), and anti-GAPDH (BBI Life Sciences, D110016-0100).

### RNA extraction, reverse transcription, and real-time polymerase chain reaction (PCR)

Total RNA was extracted from tissues using RNA extraction kit (Bioteke) following the manufacturer’s protocol. RNA was then reverse-transcribed with RT reagent kit (Takara, Japan). The cDNAs were subsequently subjected to SYBR Green-based real-time PCR analysis. GAPDH was used for normalization. Data were shown as average values ± standard error of the mean (SEM). The *p* value was calculated using the Student’s t-test. The primers used in qPCR were listed in Table S1.

### RNA sequencing

Gastric mRNA was obtained from 12-week-old *Atp4b-cre; Myc^OE^* and wild type (WT) mice. Differential gene expression was analyzed using the DESeq2 package. The list of significance was determined by setting a false discovery rate (FDR) threshold at a level of <0.05 and |log_2_FC| of more than 0.585. All differentially expressed genes were subsequently analyzed for gene ontology (GO) and pathway analysis.

### Cell culture

AGS cell line was obtained from the American Type Culture Collection (ATCC) and cultured in RPMI-1640 supplemented with 10% fetal bovine serum (Thermo), 100 U/mL penicillin, and 0.1 mg/mL streptomycin (Thermo) at 37°C in a humidified 5% CO_2_ atmosphere.

### Plasmids and transfection

Human *c-Myc* cDNA was generated by PCR and cloned into pCMV6-Entry vector with Myc-tag and flag-tag. The constructs generated were confirmed by DNA sequencing. For transient transfection, AGS cells were transfected with the jetPRIME^®^ transfection reagent (Polyplus) according to the manufacturer’s instruction. Primers used for amplification of human *c-Myc* cDNA were as follows: Sense: 5’-AGTAAAGCTTATGGATTTTTTTCGGGTAGTGGAA-3’ and antisense: 5’-ATATACGCGTCGCACAAGAGTTCCGTAG-3’.

### CCK8 assays

Cell counting kit-8 (CCK-8, Dojindo, CK04), being non-radioactive, allows sensitive colorimetric assays for the determination of the number of viable cells in cell proliferation and cytotoxicity assays. Cells that transfected for 24 hours were seeded in 96-well plates at a density of 1 × 10^4^ cells/well. After cultured for 4 hours, cells were treated with MK-2206 and rapamycin at a concentration of 10 uM and 25 uM, respectively. After cultured for 12 hours, 24 hours, 36 hours, and 48 hours, cells were incubated with CCK8 for 2 hours at 37°C. Cell proliferation was determined by measuring the optical density value at 450 nm using a microplate reader (BioTek).

### MK-2206 and rapamycin treatment

*Atp4b-cre; Myc^OE^* mice, at 7 weeks of age, were treated with two inhibitors, MK-2206 and rapamycin, 3 mice in each group. MK-2206 (MCE, HY-10358) was prepared in 10% dimethyl sulfoxide (DMSO), 40% polyethylene glycol 300 (PEG300), 5% Tween-80, and 45% saline and administered to *c-Myc* transgenic mice by oral gavage at a dose of 100 mg/kg every other day for 2 weeks. Rapamycin (MCE, HY-10219) was prepared in 10% DMSO, 40% PEG300, 5% Tween-80, and 45% saline and administered to *c-Myc* transgenic mice by intraperitoneal injection at a dose of 5 mg/kg daily for 3 weeks. Mice were then sacrificed and stomachs were harvested for further HE staining.

### Statistical analysis

All experiments were repeated at least three times. Unless otherwise indicated, data were presented as mean ± SEM and analyzed for statistical significance by Kruskal–Wallis or Mann–Whitney using GraphPad Prism 6 software or SPSS 19.0 software. *p* < 0.05 was considered to be statistically significant. *p < 0.05, **p < 0.01, ***p < 0.001, ****p < 0.0001.

### Data availability

RNA-Seq raw data have been deposited in the Gene Expression Omnibus (GEO) under accession number GEO: GSE145583.

## RESULTS

### Overexpression of *c-Myc* in mouse gastric parietal cells induces gastric tumorigenesis

**FIGURE 1:**
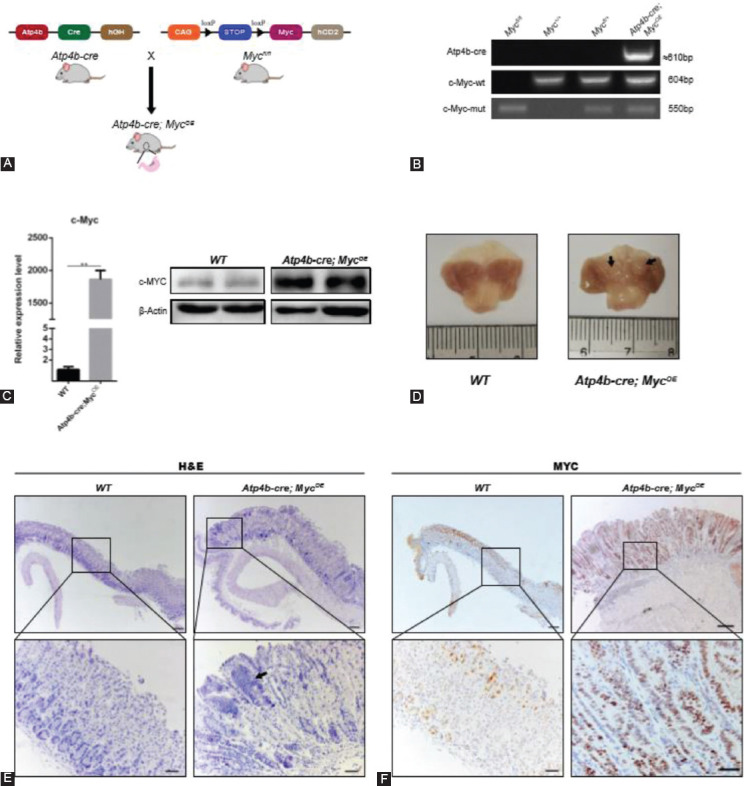
Generation of *Myc* transgenic mouse model of gastric adenoma. (A) Schematic representation of *Atp4b-cre; Myc*^OE^ mice. (B) Identification of *Atp4b-cre; Myc*^OE^ mice by polymerase chain reaction from genomic DNA. (C) Confirmation of *c-Myc* overexpression at RNA and protein level. (D) Gross pictures of *Atp4b-cre*; *Myc*^OE^ mouse stomach compared with wild type (WT) control. Arrows indicated protrusion in the corpus region. (E) Hematoxylin and eosin images (top, scale bar, 200 µm) and enlarged images (bottom, scale bar, 50 µm) of *Atp4b-cre; Myc*^OE^ and WT mouse stomachs (12 weeks of age). Arrows indicate dysplasia. (F) MYC immunohistochemistry (top, scale bar, 200 µm) and enlarged images (bottom, scale bar, 50 µm) of stomach sections from *Atp4b-cre; Myc*^OE^ and WT mice.

To investigate the role of *c-Myc* in GC, we first generated a mouse model with Cre-dependent targeted overexpression of *c-Myc*. *c-Myc*-floxed mice were crossed with *Atp4b-cre* to obtain *Atp4b-cre; Myc^OE^* mice (Figure 1A). In *Atp4b-cre; Myc^OE^* mice, *c-Myc* was specifically overexpressed in *Atp4b^+^* gastric parietal cell lineages, which are the most abundant cells in gastric mucosa. *Myc^fl/fl^* littermates of mice were used as control, referred to here as WT. After genotyping, *Atp4b-cre; Myc^OE^* mice were selected (Figure 1B). RT-qPCR and western blotting confirmed remarkable overexpression of *c-Myc* in the mouse stomach (Figure 1C). Compared with WT mice, *c-Myc* transgenic mice were viable and showed no significant alterations in weight. However, visible tumors were observed in the stomachs of *Atp4b-cre; Myc^OE^* mice (Figure 1D). Tumors were mainly occurred in the corpus of the mouse stomach. H&E staining showed abnormal hyperplasia in *c-Myc* transgenic mice (Figure 1E). By immunostaining, significant-high expression of c-MYC was confirmed in the gastric mucosa (Figure 1F). Collectively, these results demonstrate that stomach-specific overexpression of *c-Myc* can induce tumors in the mouse stomach.

### *c-Myc* transgenic mice display an age-dependent progressive gastric histopathology

**FIGURE 2:**
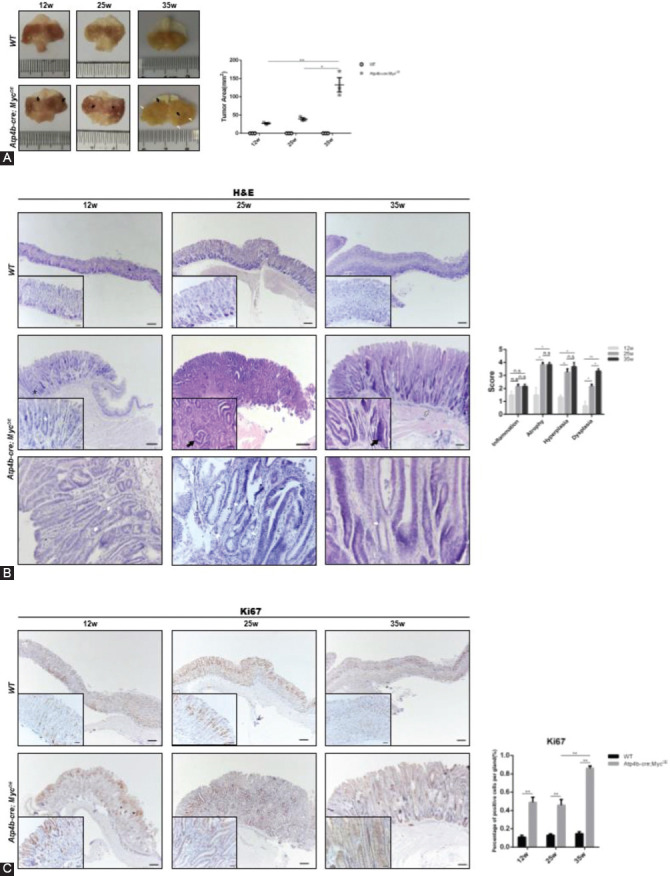
Representative gastric histopathology at different ages of *Myc* transgenic mice. (A) Gross pictures of stomachs from *Atp4b-cre; Myc*^OE^ and wild type (WT) mice at 12, 25, and 35 weeks of age. Black arrows indicated thickened gastric walls and white arrows indicated polypus. Black arrowhead indicated small tumors protruding from the mucous lining of the corpus. Tumor area (mm^2^) is quantified at right. (B) Serial hematoxylin and eosin staining (scale bar, 200 µm) and enlarged images (insets, scale bar, 50µm) of the fundic stomach mucosa from *Atp4b-cre; Myc*^OE^ and WT mice at 12, 25, and 35 weeks of age. The second row of HE pictures from *Atp4b-cre; Myc*^OE^ showed IM. White asterisk indicated atrophy with loss of parietal cells and chief cells. Black asterisk indicated hyperplasia. White arrows indicated the presence of goblet cells. Black arrows indicated dysplasia. Hollow arrow indicated inflammation. Histologic scores of fundus from *Atp4b-cre; Myc*^OE^ mice (n ≥ 3) were evaluated and showed at right. A score of 4 denotes the highest pathologic severity and a score of 0 denotes the normal condition [39]. (C) Immunohistochemistry images of Ki67 (scale bar, 200 µm) and enlarged images (insets, scale bar, 50 µm) of the fundic stomach mucosa from *Atp4b-cre; Myc*^OE^ and WT mice at 12, 25, and 35 weeks of age. Quantification of Ki67 staining is shown at right. *p < 0.05, **p < 0.01, ***p < 0.001, ****p < 0.0001.

To analyze tumorigenesis driven by *c-Myc* in the stomach, mice were sacrificed at sequential time points. At gross examination, there was obvious mass protrusion in the corpus region. Tumor area increased as the mice grew older (Figure 2A). In histology sections, we found microscopic changes in the gastric mucosal epithelium of *Atp4b-cre; Myc^OE^* mice (Figure 2B and Table S2). At 12 weeks, there was a slight loss of parietal cells and chief cells, indicating atrophy. We also observed a moderate elongation of the surface-type epithelium, which was hyperplasia. At 25 weeks, these lesions became more severe, characterized by tubule branching and infolding, cell piling up, and increased nuclear–cytoplasmic (N–C) ratio (dysplasia). At 35 weeks, tumors progressed to adenoma. Cells in the lesions displayed hyperchromatic nuclei and loss of polarity with almost a total loss of parietal cells and chief cells. Inflammatory cells extended into submucosa and mucosa. Besides, we observed different degrees of IM in mice of three ages, with the most severe IM in mice at 25 weeks (Figure 2B). The intense Ki67 staining of these lesions suggested that they were highly proliferative (Figure 2C). Taken together, our results indicate that *c-Myc* overexpression in the mouse gastric mucosa triggers a stepwise progression from hyperplasia to adenoma. Besides, gastric tumorigenesis due to *c-Myc* overexpression shows characteristics of an early onset and a long precancerous stage.

**TABLE S2:**
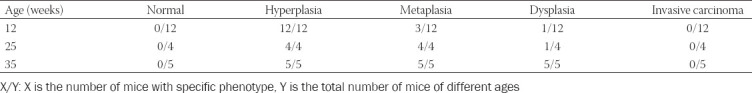
Disease progression in c-Myc transgenic mouse model

### *Atp4b-cre; Myc^OE^* transgenic mice exhibit increased intestinal characteristics and decreased gastric mucins

**FIGURE 3:**
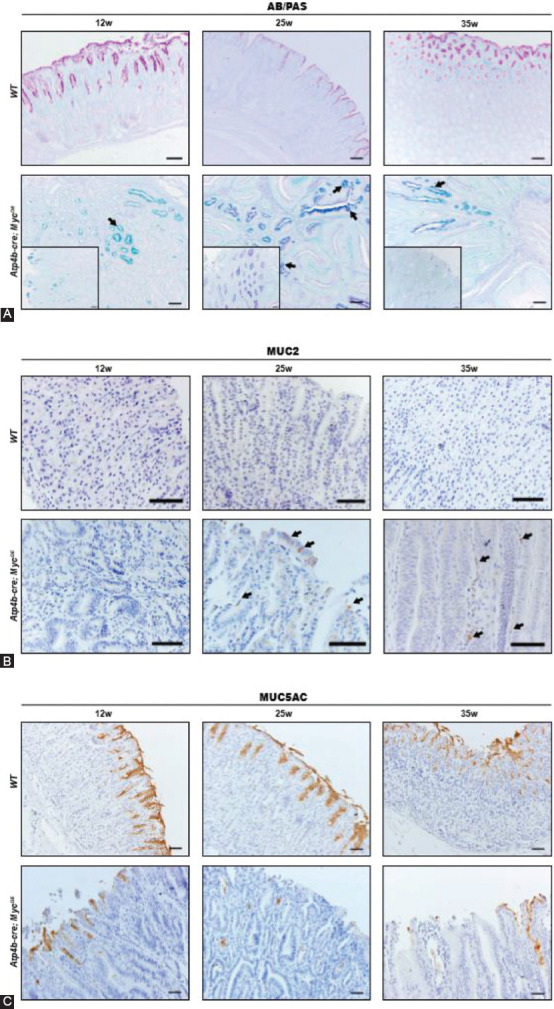
Increase of intestinal characteristics and decrease of gastric mucins. (A) Alcian blue and periodic acid-Schiff (AB/PAS)-stained sections of the fundic stomach mucosa from *Atp4b-cre; Myc*^OE^ and wild type (WT) mice at 12, 25, and 35 weeks of age. Insets showed the staining of the surface regions of mouse stomachs. Arrows indicated blue-stained goblet cells. Scale bars, 50 µm. (B) MUC2 staining of the fundic stomach mucosa from *Atp4b-cre; Myc*^OE^ and WT mice at 12, 25, and 35 weeks of age. Scale bars, 50 µm. Arrows indicate positive staining. (C) MUC5AC staining of the fundic stomach mucosa from *Atp4b-cre; Myc*^OE^ and WT mice at 12, 25, and 35 weeks of age. Scale bars, 50 µm.

To further investigate the tumor characteristics in *c-Myc* transgenic mice, we performed immunostaining for intestinal and gastric markers in gastric tissues. It is well-known that IM is a precancerous lesion of GC [40]. IM is a process of gastric epithelial cells that undergo trans-differentiation to intestinal cells, which mainly express acid mucins [40]. As shown in Figure 3A, AB/PAS staining revealed AB^+^ cells (indicating acid mucins) in *Atp4b-cre; Myc^OE^* mice and PAS^+^ cells (indicating neutral mucins) in WT mice (Figure 3A). The normal gastric mucosa specifically expresses MUC5AC, which is mainly found in the superficial epithelium [41]. MUC2 is intestinal mucin and cannot be detected in the normal gastric mucosa [41]. Notably, MUC2 immunostaining showed positive expression of MUC2 in the gastric epithelium of *c-Myc* transgenic mice compared with WT mice, while the expression of MUC5AC in *Atp4b-cre; Myc^OE^* mice was decreased as MUC5AC staining indicated (Figure 3B and C). Together, these staining results indicate IM in *Atp4b-cre; Myc^OE^* mice and suggest that *c-Myc*-driven gastric tumors share similarities with intestinal-type GC.

### Transcriptome analysis reveals that *c-Myc* promotes tumorigenesis in mice by impacting PI3K/AKT signaling

**FIGURE 4:**
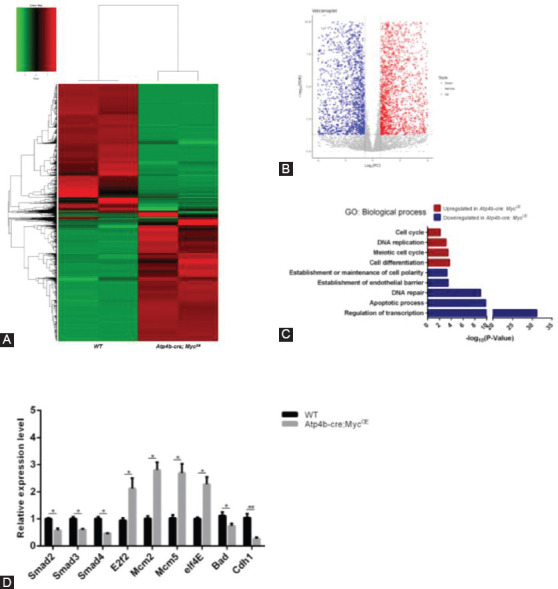
Transcriptome analysis of *c-Myc*-induced gastric tumors. (A) Heatmap summarization of differentially expressed genes associated with *c-Myc* overexpression from RNA-seq data. (B) Volcano plot showing the relative distribution of upregulated and downregulated genes related to *c-Myc* overexpression. (C) Gene ontology (GO) analysis of genes significantly up- and down-regulated in *Atp4b-cre; Myc*^OE^ mice compared with wild type mice. (D) RT-qPCR analysis of representative differentially expressed genes from RNA-seq data. *p < 0.05, **p < 0.01, ***p < 0.001.

To explore the potential mechanism underlying *c-Myc*-mediated tumor growth, we performed RNA sequencing with gastric tissues from WT and *Atp4b-cre; Myc^OE^* mice. By analyzing and comparing transcriptome data from WT and *c-Myc* transgenic mice, we identified 14930 differentially expressed genes, including 6718 upregulated genes and 8212 downregulated genes (Figure 4A and B). A subsequent gene ontology (GO) analysis of biological process terms revealed a significant enrichment of genes related to cell cycle and other cellular functions, which could be attributed to high expression of *c-Myc* and subsequent tumorigenesis (Figure 4C). RT-qPCR of several genes from GO data was performed to validate the results of RNA-seq (Figure 4D). As expected, RNA expression levels of *c-Myc* target genes (*Mcm2/Mcm5/eIF4E*) were upregulated compared with WT mice. Notably, the RNA expression of *Smad2/3/4*, which is related to regulation of transcription, was significantly downregulated in *c-Myc* transgenic mice compared with WT mice. Furthermore, the RNA expression of *Mcm2/Mcm5/E2f2*, associated with cell cycle, was significantly upregulated in *Atp4b-cre; Myc^OE^* mice.

**FIGURE 5:**
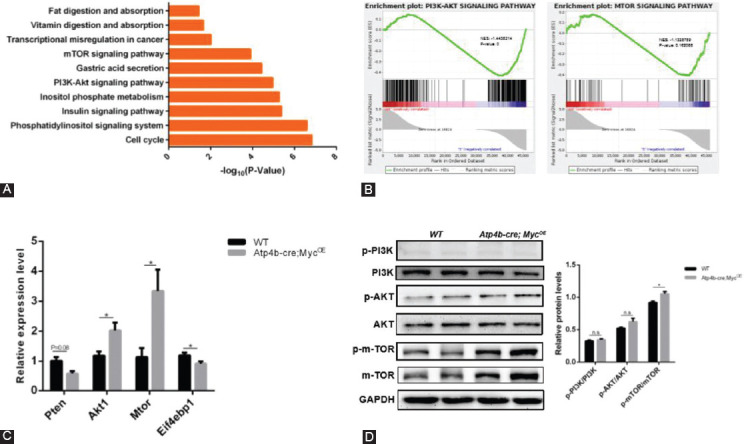
Activation of the PI3K/AKT pathway in *Atp4b-cre; Myc*^OE^ mice. (A) Pathway analysis of deregulated genes in *Atp4b-cre; Myc*^OE^ mice compared with wild type (WT) mice. (B) GSEA enrichment plots of differentially expressed genes belonging to PI3K-Akt pathway and mTOR pathway associated with *c-Myc* overexpression. (C) RT-qPCR analysis of key genes in PI3K/AKT/mTOR signaling. (D) Western blotting analysis of the indicated proteins from stomach lysates of *Atp4b-cre; Myc*^OE^ mice and WT mice. Quantification of the indicated protein levels (normalized to GAPDH) was shown at right. *p < 0.05.

A subsequent pathway analysis revealed a significant enrichment of genes related to phosphatidylinositol signaling system, inositol phosphate metabolism, PI3K-Akt pathway, and mTOR pathway (Figure 5A). Gene set enrichment analysis (GSEA) data showed that *c-Myc* overexpression enriched genes correlated with the PI3K-Akt pathway and mTOR signaling (Figure 5B). To further validate the change of this pathway, RT-qPCR was performed to examine the RNA expression level of several key genes. Akt1 and mTOR, which are main factors of PI3K-Akt pathway, exhibited higher RNA expression levels in *Atp4b-cre; Myc^OE^* mice compared with WT mice, while Pten, an inhibitory factor of this pathway, was slightly downregulated (Figure 5C). The western blotting analysis confirmed that *Atp4b-cre; Myc^OE^* mice exhibited profound increases of p-mTOR/mTOR and a slight increase of p-AKT/AKT (*p* > 0.05) compared with WT mice (Figure 5D), while p-PI3K/PI3K did not show a significant difference between two groups. Taken together, these results indicate that *c-Myc* may promote tumorigenesis of mice through AKT/mTOR signaling.

### Inhibition of the AKT/mTOR pathway attenuates proliferation in *c-Myc* overexpressing GC cell line and inhibits the initiation of gastric tumorigenesis *in vivo*

**FIGURE 6:**
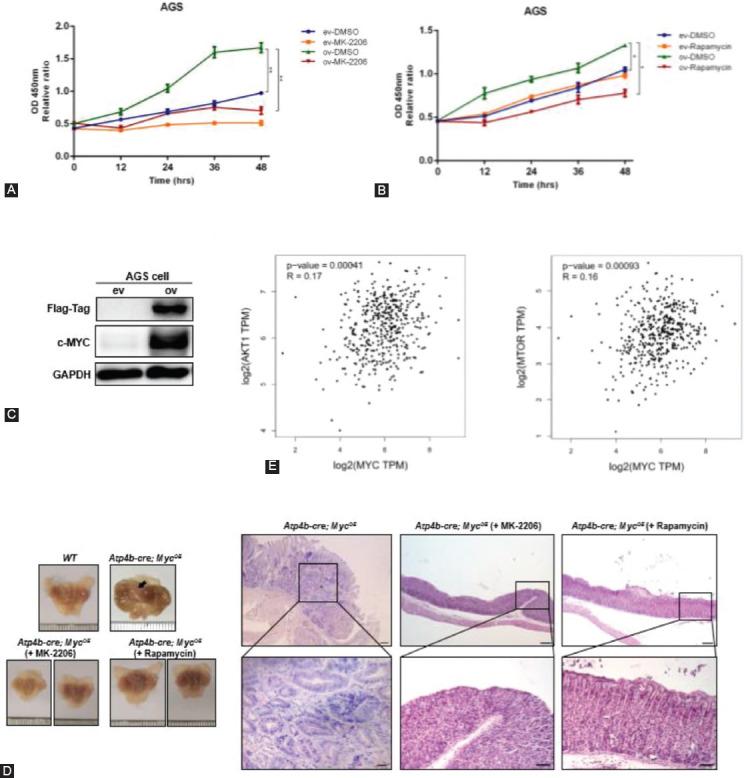
Blocking AKT/mTOR pathway decreased proliferation of AGS cells overexpressing *c-Myc* and inhibited onset of gastric tumorigenesis in *c-Myc* transgenic mice. (A) CCK8 assay of AGS cells transfected with empty vector (ev) and *c-Myc* plasmids (ov) after treatment with the AKT inhibitor MK-2206 and solvent dimethyl sulfoxide (DMSO). (B) CCK8 assay of AGS cells transfected with empty vector (ev) and *c-Myc* plasmids (ov) after treatment with the mTOR inhibitor rapamycin and solvent DMSO. (C) Western blotting analysis of the indicated proteins from AGS cell lysates (ev & ov). (D) Hematoxylin and eosin staining (scale bar, 200 µm) and enlarged images (scale bar, 50 µm) of the fundic stomach mucosa from *Atp4b-cre; Myc*^OE^ mice treated with MK-2206, rapamycin, and vehicle. Gross pictures of stomachs were shown at the left. Arrow indicated thickened gastric wall (white). (E) Gene expression correlation analysis of *c-Myc* and *Akt1/Mtor* in human gastric cancer samples from TCGA datasets. Data analysis was performed on the GEPIA platform (http://gepia.cancer-pku.cn/detail.php) and used the TCGA-STAD datasets. *p < 0.05, **p < 0.01, ***p < 0.001.

To further elucidate the causal link between *c-Myc* and the AKT/mTOR pathway in GC, we transfected *c-Myc* plasmids into AGS cells and then treated these cells with an AKT inhibitor (MK-2206) or an mTOR inhibitor (rapamycin). As shown by CCK8 assays, overexpression of *c-Myc* in AGS cells (Figure 6C) promoted cell proliferation, while treatment of MK-2206 or rapamycin significantly suppressed cell proliferation (Figure 6A and B). Moreover, we also treated *Atp4b-cre; Myc^OE^* mice with MK-2206 and rapamycin to test *in vivo* effect of inhibiting AKT/mTOR pathway in gastric tumorigenesis. Compared with the control group, *Atp4b-cre; Myc^OE^* mice treated with MK-2206 and rapamycin did not present any abnormal changes and typical features of hyperplasia or intestinal metaplasia (Figure 6D). Thus, blocking AKT/mTOR signaling may inhibit the initiation of gastric tumorigenesis. In addition, analysis of TCGA datasets indicated that the *c-Myc* expression was positively correlated with *Akt1* and *Mtor* expression in GC, respectively (Figure 6E). Collectively, these results suggest that the oncogenic role of *c-Myc* is mediated through the activation of AKT/mTOR signaling and blocking AKT/mTOR signaling may be helpful to inhibit or postpone the onset of gastric tumors.

## DISCUSSION

Although the incidence of GC has decreased in recent years attributed to the improvement of sanitary conditions and eating habits, there were over one million new cases of GC and more than 780,000 deaths due to GC worldwide in 2018 [1]. GC has a long and asymptomatic precancerous phase, which mainly includes intestinal metaplasia and dysplasia. Therefore, the study of precancerous stages of GC and identification of the drivers for this process are of great significance to prevent and to diagnose GC earlier. Previous studies suggest that overexpression of *c-Myc* is associated with malignant progress and poor survival in GC patients [19,20]. It is also reported that significantly higher MYC expression was observed in IM samples than gastritis samples from cancer-free individuals and this may facilitate tumor initiation [42]. However, the causal role of *c-Myc* in induction of GC has been unknown. Our work presents a definite answer regarding the sufficient function of *c-Myc* in causing the gastric epithelial cells to undergo serial steps of tumorigenesis from an early precancerous phase, including IM and dysplasia to the formation of adenoma. Our findings add *c-Myc* as a causal oncogene to the existing list of GC drivers, which includes Notch, hedgehog, *CDH1*, and *TP53* [7,43-45].

By the establishment of a conditional transgenic mouse model, we show that gastric adenoma induced by *c-Myc* overexpression is achieved through activation of AKT/mTOR signaling. Our findings are in agreement with many other studies. The AKT/mTOR pathway is a canonical pathway involved in the regulation of multiple cellular functions, including cell proliferation, apoptosis, and metabolism. Aberrations in the PI3K/AKT/mTOR pathway in head and neck squamous cell carcinoma (HNSCC) were associated with malignant characteristics [46]. Activation of the AKT/mTOR pathway is often seen in oral squamous cell carcinoma [47], skin cancer [48,49], and RCC [50]. It has also been reported that the AKT/mTOR pathway plays a crucial role in the development of GC [51,52]. Our results showed that expression levels of AKT and mTOR are significantly increased in *c-Myc* transgenic mice, and inhibition of AKT and mTOR can significantly decrease cell proliferation in AGS cells overexpressing *c-Myc* and inhibit or postpone the onset of gastric tumors *in vivo*. Importantly, our experiments demonstrate not only that *c-Myc* can be a driver for gastric adenoma but also that the AKT/mTOR pathway could be the underlying mechanism of gastric tumorigenesis caused by *c-Myc* overexpression.

It is worth pointing out that our study also shows an increased copy number of *c-Myc* gene can prompt the gastric tumorigenesis of transgenic mice toward a faster and more severe way. Based on our observation, 14-week-old *Atp4b-cre; Myc^fl/fl^* mice exhibit submucosal invasion, while *Atp4b-cre; Myc^OE^* (*Atp4b-cre; Myc^fl/+^*) mice do not at the same age. Similarly, in human GC, it is reported that increased *Myc* copy number is associated with a late-onset, intestinal-type cancer and the presence of distant metastasis [12]. Whether and when *Atp4b-cre*; *Myc^fl/fl^* mice exhibit distant metastasis needs further investigation.

Taken together, we generated a novel autochthonous transgenic mouse model of gastric adenoma that is generally useful for studying the initiation and progression of GC. It provides a new platform to further study the roles of more genes involved in GC through combining with mutations in other genes. It will facilitate our better understanding of the development of early GC and shed light on the molecular mechanisms by which *c-Myc* affects the development and progression of GC. More importantly, it will aid the clinical detection and therapeutic strategies for intervention at precancerous stages of GC so to improve patient survival.

## References

[1] Bray F, Ferlay J, Soerjomataram I, Siegel RL, Torre LA, Jemal A (2018). Global cancer statistics 2018:GLOBOCAN estimates of incidence and mortality worldwide for 36 cancers in 185 countries. CA Cancer J Clin.

[2] Jemal A, Bray F, Center MM, Ferlay J, Ward E, Forman D (2011). Global cancer statistics. CA Cancer J Clin.

[3] Laurén PA (1965). The two histological main types of gastric carcinoma:Diffuse and so-called intestinal-type carcinoma. An attempt at a histo-clinical classification. Acta Pathol Microbiol Scand.

[4] Liu W, Pan HF, Wang Q, Zhao ZM (2018). The application of transgenic and gene knockout mice in the study of gastric precancerous lesions. Pathol Res Pract.

[5] Correa P (1992). Human gastric carcinogenesis:A multistep and multifactorial process First American cancer society award lecture on cancer epidemiology and prevention. Cancer Res.

[6] Chinwalla AT, Cook LL, Delehaunty KD, Fewell GA, Fulton LA, Fulton RS (2002). Initial sequencing and comparative analysis of the mouse genome. Nature.

[7] Poh AR, O'Donoghue RJ, Ernst M, Putoczki TL (2016). Mouse models for gastric cancer:Matching models to biological questions. J Gastroenterol Hepatol.

[8] Calcagno DQ, Freitas VM, Leal MF, de Souza CR, Demachki S, Montenegro R (2013). MYC, FBXW7 and TP53 copy number variation and expression in gastric cancer. BMC Gastroenterol.

[9] Choi JS, Seo J, Jung EJ, Kim EJ, Lee GK, Kim WH (2012). c-MYC amplification in mucinous gastric carcinoma:A possible genetic alteration leading to deeply invasive tumors. Anticancer Res.

[10] Zhang F, Li K, Yao X, Wang H, Li W, Wu J (2019). A miR-567-PIK3AP1-PI3K/AKT-c-Myc feedback loop regulates tumour growth and chemoresistance in gastric cancer. EBioMedicine.

[11] Gao S, Chen M, Wei W, Zhang X, Zhang M, Yao Y (2018). Crosstalk of mTOR/PKM2 and STAT3/c-Myc signaling pathways regulate the energy metabolism and acidic microenvironment of gastric cancer. J Cell Biochem.

[12] de Souza CR, Leal MF, Calcagno DQ, Sozinho EK, Borges Bdo N, Montenegro RC (2013). MYC deregulation in gastric cancer and its clinicopathological implications. PLoS One.

[13] Raiol LC, Silva EC, da Fonseca DM, Leal MF, Guimaraes AC, Calcagno DQ (2008). Interrelationship between MYC gene numerical aberrations and protein expression in individuals from northern Brazil with early gastric adenocarcinoma. Cancer Genet Cytogenet.

[14] Han S, Kim HY, Park K, Cho HJ, Lee MS, Kim HJ (1999). c-Myc expression is related with cell proliferation and associated with poor clinical outcome in human gastric cancer. Journal of Korean Medical Science.

[15] Milne AN, Sitarz R, Carvalho R, Carneiro F, Offerhaus GJ (2007). Early onset gastric cancer:On the road to unraveling gastric carcinogenesis. Curr Mol Med.

[16] Panani AD (2008). Cytogenetic and molecular aspects of gastric cancer:clinical implications. Cancer Lett.

[17] Xu AG, Li SG, Liu JH, Gan AH (2001). Function of apoptosis and expression of the proteins Bcl-2, p53 and C-myc in the development of gastric cancer. World J Gastroenterol.

[18] Yang GF, Deng CS, Xiong YY, Gong LL, Wang BC, Luo J (2004). Expression of nuclear factor-kappa B and target genes in gastric precancerous lesions and adenocarcinoma:Association with Helicobactor pylori cagA (+) infection. World J Gastroenterol.

[19] Wang X, Liu Y, Shao D, Qian Z, Dong Z, Sun Y (2016). Recurrent amplification of MYC and TNFRSF11B in 8q24 is associated with poor survival in patients with gastric cancer. Gastric Cancer.

[20] Khaleghian M, Shakoori A, Razavi AE, Azimi C (2015). Relationship of amplification and expression of the C-MYC gene with survival among gastric cancer patients. Asian Pac J Cancer Prev.

[21] Lan J, Xiong YY, Lin YX, Wang BC, Gong LL, Xu HS (2003). Helicobacter pylori infection generated gastric cancer through p53-Rb tumor-suppressor system mutation and telomerase reactivation. World J Gastroenterol.

[22] Zhang GX, Gu YH, Zhao ZQ, Xu SF, Zhang HJ, Wang HD (2004). Coordinate increase of telomerase activity and c-Myc expression in Helicobacter pylori-associated gastric diseases. World J Gastroenterol.

[23] Elbadawy M, Usui T, Yamawaki H, Sasaki K (2019). Emerging roles of C-Myc in cancer stem cell-related signaling and resistance to cancer chemotherapy:A potential therapeutic target against colorectal cancer. Int J Mol Sci.

[24] Agarwal E, Altman BJ, Seo JH, Ghosh JC, Kossenkov AV, Tang HY (2019). Myc-mediated transcriptional regulation of the mitochondrial chaperone TRAP1 controls primary and metastatic tumor growth. J Biol Chem.

[25] Shroff EH, Eberlin LS, Dang VM, Gouw AM, Gabay M, Adam SJ (2015). MYC oncogene overexpression drives renal cell carcinoma in a mouse model through glutamine metabolism. Proc Natl Acad Sci U S A.

[26] Sikora K, Chan S, Evan G, Gabra H, Markham N, Stewart J (1987). c-myc oncogene expression in colorectal cancer. Cancer.

[27] Erisman MD, Rothberg PG, Diehl RE, Morse CC, Spandorfer JM, Astrin SM (1985). Deregulation of c-myc gene expression in human colon carcinoma is not accompanied by amplification or rearrangement of the gene. Mol Cell Biol.

[28] Cancer Genome Atlas Network. Comprehensive molecular characterization of human colon and rectal cancer (2012). Nature.

[29] Ba M, Long H, Yan Z, Wang S, Wu Y, Tu Y (2018). BRD4 promotes gastric cancer progression through the transcriptional and epigenetic regulation of c-MYC. J Cell Biochem.

[30] Zhang L, Hou Y, Ashktorab H, Gao L, Xu Y, Wu K (2010). The impact of C-MYC gene expression on gastric cancer cell. Mol Cell Biochem.

[31] Chen M, Ling T, Wang L, Zou X, Gao S (2018). Su1964 crosstalk of Mtor/Hif-1A/Pkm2 and Stat3/C-Myc signaling pathways regulate the energy metabolism and acidic microenvironment of gastric cancer. Gastroenterology.

[32] Liu H, Liu N, Zhao Y, Zhu X, Wang C, Liu Q (2019). Oncogenic USP22 supports gastric cancer growth and metastasis by activating c-Myc/NAMPT/SIRT1-dependent FOXO1 and YAP signaling. Aging.

[33] Xu TP, Ma P, Wang WY, Shuai Y, Wang YF, Yu T (2019). KLF5 and MYC modulated LINC00346 contributes to gastric cancer progression through acting as a competing endogeous RNA and indicates poor outcome. Cell Death Differ.

[34] Choi W, Kim J, Park J, Lee DH, Hwang D, Kim JH (2018). YAP/TAZ initiates gastric tumorigenesis via upregulation of MYC. Cancer Res.

[35] Ellis L, Ku S, Li Q, Azabdaftari G, Seliski J, Olson B (2016). Generation of a C57BL/6 MYC-driven mouse model and cell line of prostate cancer. Prostate.

[36] Xu P, Widmer G, Wang Y, Ozaki LS, Alves JM, Serrano MG (2004). The genome of *Cryptosporidium hominis*. Nature.

[37] Zhao Z, Hou N, Sun Y, Teng Y, Yang X (2010). Atp4b promoter directs the expression of Cre recombinase in gastric parietal cells of transgenic mice. J Genet Genomics.

[38] Calado DP, Sasaki Y, Godinho SA, Pellerin A, Kochert K, Sleckman BP (2012). The cell-cycle regulator c-Myc is essential for the formation and maintenance of germinal centers. Nat Immunol.

[39] Rogers AB (2012). Histologic scoring of gastritis and gastric cancer in mouse models. In:Houghton J, editor. Helicobacter Species:Methods and Protocols.

[40] Companioni O, Sanz-Anquela JM, Pardo ML, Puigdecanet E, Nonell L, Garcia N (2017). Gene expression study and pathway analysis of histological subtypes of intestinal metaplasia that progress to gastric cancer. PLoS One.

[41] Reis CA, David L, Correa P, Carneiro F, Sobrinho-Simões M (1999). Intestinal metaplasia of human stomach displays distinct patterns of mucin (MUC1, MUC2, MUC5AC, and MUC6) expression. Cancer Res.

[42] Silva TC, Leal MF, Calcagno DQ, de Souza CR, Khayat AS, dos Santos NP (2012). hTERT, MYC and TP53 deregulation in gastric preneoplastic lesions. BMC Gastroenterol.

[43] Penon D, Cito L, Giordano A (2014). Novel findings about management of gastric cancer:A summary from 10th IGCC. World J Gastroenterol.

[44] Cancer Genome Atlas Research Network. Comprehensive molecular characterization of gastric adenocarcinoma (2014). Nature.

[45] El-Zaatari M, Kao JY, Tessier A, Bai L, Hayes MM, Fontaine C (2013). Gli1 deletion prevents Helicobacter-induced gastric metaplasia and expansion of myeloid cell subsets. PLoS One.

[46] Broek RV, Mohan S, Eytan DF, Chen Z, Van Waes C (2015). The PI3K/Akt/mTOR axis in head and neck cancer:Functions, aberrations, cross-talk, and therapies. Oral Dis.

[47] Chaisuparat R, Limpiwatana S, Kongpanitkul S, Yodsanga S, Jham BC (2016). The Akt/mTOR pathway is activated in verrucous carcinoma of the oral cavity. J Oral Pathol Med.

[48] Zou Y, Ge M, Wang X (2017). Targeting PI3K-AKT-mTOR by LY3023414 inhibits human skin squamous cell carcinoma cell growth *in vitro* and *in vivo*. Biochem Biophys Res Commun.

[49] Hafner C, Landthaler M, Vogt T (2010). Activation of the PI3K/AKT signalling pathway in non-melanoma skin cancer is not mediated by oncogenic PIK3CA and AKT1 hotspot mutations. Exp Dermatol.

[50] Guo H, German P, Bai S, Barnes S, Guo W, Qi X (2015). The PI3K/AKT pathway and renal cell carcinoma. J Genet Genomics.

[51] Tapia O, Riquelme I, Leal P, Sandoval A, Aedo S, Weber H (2014). The PI3K/AKT/mTOR pathway is activated in gastric cancer with potential prognostic and predictive significance. Virchows Arch.

[52] Ying J, Xu Q, Liu B, Zhang G, Chen L, Pan H (2015). The expression of the PI3K/AKT/mTOR pathway in gastric cancer and its role in gastric cancer prognosis. Onco Targets Ther.

